# Cancer-Associated Stromal Cells Promote the Contribution of MMP2-Positive Bone Marrow-Derived Cells to Oral Squamous Cell Carcinoma Invasion

**DOI:** 10.3390/cancers14010137

**Published:** 2021-12-28

**Authors:** May Wathone Oo, Hotaka Kawai, Kiyofumi Takabatake, Qiusheng Shan, Htoo Shwe Eain, Shintaro Sukegawa, Keisuke Nakano, Hitoshi Nagatsuka

**Affiliations:** 1 Department of Oral Pathology and Medicine, Graduate School of Medicine, Dentistry and Pharmaceutical Sciences, Okayama University, Okayama 700-8525, Japan; p1qq7mbu@s.okayama-u.ac.jp (M.W.O.); gmd422094@s.okayama-u.ac.jp (K.T.); hrbmushanqiusheng@163.com (Q.S.); pmp61kpp@s.okayama-u.ac.jp (H.S.E.); gouwan19@gmail.com (S.S.); keisuke1@okayama-u.ac.jp (K.N.); jin@okayama-u.ac.jp (H.N.); 2 Department of Oral and Maxillofacial Surgery, Graduate School of Medicine, Dentistry and Pharmaceutical Sciences, Okayama University, Okayama 700-8525, Japan; 3 Department of Oral and Maxillofacial Reconstructive Surgery, Graduate School of Medicine, Dentistry and Pharmaceutical Sciences, Okayama University, Okayama 700-8525, Japan; 4 Department of Oral and Maxillofacial Surgery, Kagawa Prefectural Central Hospital, Takamatsu 760-0065, Japan

**Keywords:** oral squamous cell carcinoma invasion, patient-derived stromal cell xenograft (PDSX), bone marrow-derived cells (BMDCs), MMP2, stromal factor IL-6, stromal factor IL1B

## Abstract

**Simple Summary:**

Based on its invasiveness, oral squamous cell carcinoma (OSCC) shows two different subtypes: less-invasive verrucous squamous carcinoma (VSCC) or highly invasive squamous cell carcinoma (SCC). The stromal component influences OSCC progression and invasion. On the other hand, bone marrow-derived cells (BMDCs) are recruited into tumors and involved in tumor development. We hypothesized that stromal factors might also affect the relation of BMDCs and tumor invasion. We established the OSCC models transplanted with stromal cells from VSCC and SCC, and we compared the potential stromal factors of VSCC and SCC for the involvement of BMDCs in tumor invasion. Our study showed that stromal factors IL6 and IL1B might promote the contribution of MMP-2 positive BMDCs to OSCC invasion.

**Abstract:**

Tumor stromal components contribute to tumor development and invasion. However, the role of stromal cells in the contribution of bone marrow-derived cells (BMDCs) in oral squamous cell carcinoma (OSCC) invasion is unclear. In the present study, we created two different invasive OSCC patient-derived stroma xenografts (PDSXs) and analyzed and compared the effects of stromal cells on the relation of BMDCs and tumor invasion. We isolated stromal cells from two OSCC patients: less invasive verrucous OSCC (VSCC) and highly invasive conventional OSCC (SCC) and co-xenografted with the OSCC cell line (HSC-2) on green fluorescent protein (GFP)-positive bone marrow (BM) cells transplanted mice. We traced the GFP-positive BM cells by immunohistochemistry (IHC) and detected matrix metalloproteinase 2 (MMP2) expression on BM cells by double fluorescent IHC. The results indicated that the SCC-PDSX promotes MMP2-positive BMDCs recruitment to the invasive front line of the tumor. Furthermore, microarray analysis revealed that the expressions of interleukin 6; *IL-6* mRNA and interleukin 1 beta; *IL1B* mRNA were higher in SCC stromal cells than in VSCC stromal cells. Thus, our study first reports that IL-6 and IL1B might be the potential stromal factors promoting the contribution of MMP2-positive BMDCs to OSCC invasion.

## 1. Introduction

Head and neck cancer is the sixth most common cancer globally. Oral squamous cell carcinoma (OSCC) is a common malignant neoplasm of the head and neck region [1,2] which is caused by smoking, snuff, betel quid chewing, alcohol consumption, and viral infection [3]. The treatment for OSCC is mainly based on the extent of the cancer and includes surgery, chemotherapy, and radiotherapy. Recently, technological innovations have helped in the surgical treatment of advanced-stage OSCC and enhanced the efficacy of chemotherapy [4]. The prognosis of OSCC differs according to the subtype. OSCC shows two macroscopic subtypes with the difference in clinical invasion pattern: the endophytic type and the exophytic type [5,6]. The endophytic type is the conventional OSCC (SCC) and shows inward growth, high invasion, and occasionally metastasis. On the other hand, the exophytic type is the verrucous OSCC (VSCC), showing the expensive growth with low invasion, metastasis, and relatively better prognosis than SCC [7].

Tumor tissue is not a solo construction of the tumor cells but is constructed with stromal cells, immune cells, secreted factors, and extracellular matrix proteins, collectively known as the tumor microenvironment (TME) [8]. A recent study revealed that the stroma components influence the biological characteristics of tumor parenchyma of OSCC subtypes and support the cancer progression, invasion, and metastasis [9,10]. In addition, bone marrow-derived cells (BMDCs) infiltrate into the TME and play a role in tumor development [11,12]. BMDCs contribute as various cells in TME such as αSMA-positive cancer-associated fibroblasts (CAFs) [13], CD34-positive tumor-associated endothelial cells (TECs) [14], and CD11b-positive tumor-associated macrophages (TAMs) [15] and facilitate tumorigenesis, invasion, and metastasis. However, the involvement of BMDCs in the invasive character of OSCC subtypes is still unclear.

The local invasion of the tumor into the adjacent tissue was initiated by the activation of signaling pathways essential for controlling cytoskeletal dynamics in tumor cells and the turnover of the extracellular matrix and cell adhesion [16]. Matrix metalloproteinases (MMPs) contribute to tumor invasion and metastasis through the proteolysis of structural extracellular matrix proteins. In OSCC, the expression of MMP9 and MMP2 in tumor and stromal cells defined the invasiveness of oral cancer [17]. However, no study is available on whether BMDCs are involved in OSCC invasion via MMPs expression.

In this study, we aimed to investigate the relation of BMDCs and the invasiveness of OSCC subtypes. Thus, we used green fluorescent protein (GFP)-positive bone marrow transplanted (BMT) nude mice to trace the bone marrow (BM) cells. Then, OSCC patient-derived stromal cell xenografts (PDSXs) by co-transplanting the stromal cells isolated from patients with different OSCC subtypes: SCC and VSCC with OSCC cell line (HSC-2) to the BMT nude mice, and we evaluated the infiltration of BMDCs and the expression of MMP9 and MMP2 in the invasion front line of the tumor. Furthermore, microarray data and bioinformatics analyses were performed to clarify the potential role of stromal cells in the relation of BMDCs and OSCC invasion. Our findings may provide insights into the role of stroma in the crosstalk of BMDCs and the invasive character of OSCC.

## 2. Materials and Methods

### 2.1. Mice and Cell Line

Female nude mice (BALB/c-nu/nu and C57BL/6-BALB/c-nu/nu-GFP Tg mice) purchased from Shimizu Laboratory Suppliers were housed under pathogen-free conditions. Human OSCC cell line (HSC-2) was purchased from JRCB Cell Bank. Cells were grown in minimum essential medium-alpha (α-MEM) (Life Technologies, Carlsbad, CA, USA) with 10% fetal bovine serum (FBS) (Life Technologies, Carlsbad, CA, USA) and 1% antibiotic/antimycotic (Life Technologies, Carlsbad, CA, USA) in a humidified atmosphere with 5% CO_2_. Patient-derived stromal cells were isolated from patients with less-invasive verrucous OSCC (VSCC) and highly invasive conventional OSCC (SCC) [9]. The isolated stromal cells were maintained in α-MEM (Life Technologies, Carlsbad, CA, USA) with 10% FBS (Life Technologies, Carlsbad, CA, USA) and 1% antibiotic/antimycotic (Life Technologies, Carlsbad, CA, USA) in a humidified atmosphere with 5% CO_2_. This study complied with the Ethics Committee of Okayama University (approval number: 1703-042).

### 2.2. Bone Marrow Transplantation

GFP-positive total bone marrow (BM) cells (approximately 1.0 × 10^7^ cells/0.2 mL) were used for BMT, according to a previously described standard protocol [12]. Briefly, BM cells were collected from GFP nude mice and suspended within the Hanks’ balanced salt solution (HBSS) (Life Technologies, Carlsbad, CA, USA). Meanwhile, 8-week-old female nude recipient mice were subjected to 8 Gy of lethal whole-body irradiation, and BM cells were injected into the tail vein.

### 2.3. Tumor/Stroma Co-Transplantation

Tumor transplantation was subjected to the mice after 4 weeks of BMT. Two types of OSCC xenograft: VSCC and SCC (*n* = 5 for each), were created. HSC-2 tumor cells were co-transplanted together with stromal cells isolated from patients with VSCC or SCC. The mixtures of tumor cells and stromal cells were prepared at a ratio of 1:3 (1.0 × 10^6^ HSC-2 cells and 3.0 × 10^6^ stromal cells) and subcutaneously injected to the head [10]. At 28 days, all mice were euthanized, and the specimens were harvested for analysis.

### 2.4. Tissue Processing for Histological Analysis

For the preparation of the formalin-fixed paraffin-embedded sections, the harvested tumor tissues were fixed in 4% paraformaldehyde for 12 h and decalcified in 10% EDTA at 4 °C for 14 days. Then, the samples were dehydrated and embedded in paraffin. Serial sections (3 µm) were prepared. Sections were stained with hematoxylin and eosin (H&E), IHC, and fluorescent IHC.

### 2.5. Immunohistochemistry

To enhance the GFP, a goat monoclonal anti-GFP antibody (1:500, ab6673, Abcam, Cambridgeshire, UK) was employed. For the detection of MMP2 and MMP9, a rabbit polyclonal anti-MMP2 antibody (1:200, GTX104577, GeneTex, CA, USA) and a rabbit polyclonal anti-MMP9 antibody (1:500, ab38898, Abcam, Cambridgeshire, UK) were used respectively. Paraffin-embedded tissue sections were deparaffinized in a series of xylene for 15 min, rehydrated in graded ethanol solutions, and incubated in hydrogen peroxide methanol solution (3% H_2_O_2_) for 30 min to quench endogenous peroxidases. Then, antigen retrieval was performed by microwave heating in 0.01 mol/L sodium citrate buffer (pH 6.0) at 100 °C for 1 min. Following antigen retrieval, sections were incubated in 10% normal serum blocking solution for 15 min at room temperature (RT) in a humidified chamber, followed by incubation with primary antibodies at 4 °C overnight. For GFP detection, the secondary biotinylated antibody was applied for 30 min at RT, followed by incubation with the avidin-biotin complex (Vector Lab., Burlingame, CA, USA) for 30 min at RT. For MMP2 and MMP9 detection, the sections were incubated with horseradish peroxide (HRP)-conjugated secondary antibodies for 1 h at RT. Color development was performed with 3,3′-diaminobenzidine (DAB) (Histofine DAB substrate; Nichirei, Tokyo, Japan) and counterstained with Myer’s hematoxylin. Staining results were observed with an optical microscope (BX53, Olympus, Tokyo, Japan).

### 2.6. Double-Fluorescent IHC

Double-fluorescent IHC for GFP/MMP2 was performed. After the tissue sections were deparaffinized and rehydrated, the antigens were retrieved by microwave heating in 0.01 mol/L sodium citrate buffer (pH 6.0) at 100 °C for 1 min. Then, sections were incubated in blocking ACEÒ (DS Pharma Biomedical, Japan) solution for 20 min at RT in a humidified chamber, followed by incubation with primary antibodies as described above at 4 °C overnight. The secondary antibody application was performed using anti-goat IgG Alexa Flour 594 (Donkey, ref: A11058, Thermo, MA, USA) for GFP and anti-rabbit Alexa Flour 488 (Chicken, ref: A21441, Thermo, MA, USA) at a dilution of 1:100 for 1 h at RT. The sections were then stained with 0.2 g/mL of DAPI (Dojindo Laboratories, Kumamoto, Japan). The staining results were observed with All-in-One BZ-X700 fluorescence microscope (Keyence, Osaka, Japan).

### 2.7. Microarray and Bioinformatics Analyses

The stromal cells from VSCC and SCC were used to conduct the microarray analysis. The data are available in NCBI GEO (GSE164374) (the data are currently private and are scheduled to be released on January 1, 2024). Differentially expressed genes (DEGs) of VSCC and SCC were compared, and the 3-fold standard deviation (3SD) (logFC > 3) was considered as the cut-off value. Biological processes of the DEGs were analyzed using DAVID Bioinformatics Resources (6.8). Bubble plot presentation of biological process enrichment analysis was performed by R (3.6.2). A false discovery rate FDR > 0.05 was considered as the cut-off value. The top 10 hub genes were analyzed using a protein-to-protein interaction (PPI) using SRING: functional protein association networks (11.5) and Cytoscape 3.7.2 (cytohubba). A combined score > 0.4 was considered as the cut-off value, and the hub genes were selected according to the degree. Finally, the hub genes that were differentially expressed in the VSCC and SCC stromal cells were presented in the heat map using Microsoft Excel.

### 2.8. Quantification and Statistical Analysis

For quantification, we obtained an average of 5 randomly captured images per mouse (×400 magnification, *n* = 5) of the stromal area of the periphery of the tumor (skin and bone sides). The counting was performed using ImageJ (NIH, v1.52a). All statistical analyses were conducted using GraphPad Prism 9.1.1. Student’s *t*-test, 2-tailed for independent samples with equal variances, was used to compare two groups. Differences were considered significant at *p* < 0.05. Data are presented as the means ± standard deviation (SD).

## 3. Results

### 3.1. Stroma Architecture Is Different between VSCC- and SCC-PDSX Models

**Figure 1:**
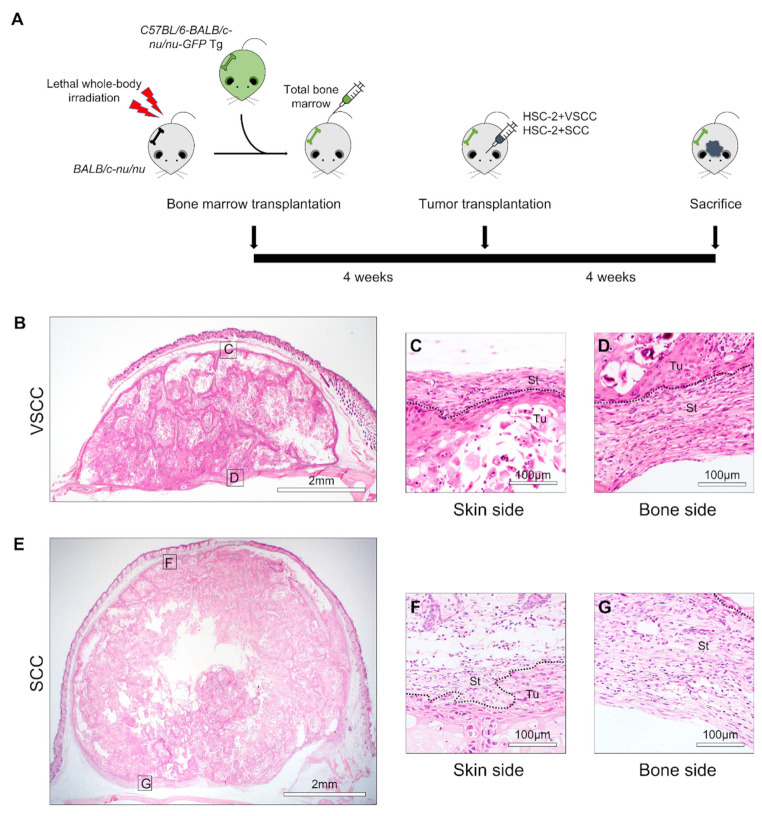
Histological characterization of VSCC-PDSX and SCC-PDSX: (**A**) Illustration of the establishment of OSCC-PDSX models. Bone marrow transplantation (BMT) with GFP-positive bone marrow cells is performed on lethal whole-body radiated nude mice. After 4 weeks, tumor transplantation is performed which allows tumors to grow for 4 weeks. Then, mice are sacrificed, and tumor tissues are harvested. (**B**) Representative images of hematoxylin and eosin (H&E) staining of VSCC-PDSX; (**C**,**D**) High magnified images of VSCC-PDSX (**C**) Skin side, (**D**) Bone; (**E**) Representative images of hematoxylin and eosin (H&E) staining of SCC-PDSX; (**F**,**G**) High magnified images of VSCC-PDSX (**F**) Skin side, (**G**) Bone side. Dotted lines represent the boundary of the tumor (Tu) and the stroma (St). VSCC, verrucous oral squamous cells carcinoma; SCC, conventional oral squamous cell carcinoma, PDSX, patient-derived stromal cells xenograft; OSCC, oral squamous cell carcinoma.

To investigate the character of tumor stroma in different subtypes of OSCC, we established the VSCC and SCC tumor models: patient-derived stromal cells were xenografted together with HSC-2 on BMT nude mice (Figure 1A).

The VSCC tumor was composed of two portions: tumor and stroma (Figure 1B). The tumor cells showed well-differentiated squamous cell carcinoma and a small area of necrosis in the central area. The stroma surrounded the tumor, and stroma tissue in the center of the tumor presented as thin and strand-like structures. The stroma and tumor area were well demarcated in the periphery area (skin and bone), and stromal tissue consisted of fibroblasts, blood vessels, and extracellular matrix (Figure 1C,D). It is also infiltrated with inflammatory cells such as neutrophils, macrophages, and dendritic cells.

The SCC tumor contains the tumor and stromal tissues (Figure 1E). The tumor cells were well-differentiated squamous cell carcinoma, and the central area showed relatively more extensive necrosis than VSCC. Unlike VSCC, SCC showed island-like small stroma in the center, and a stroma part in the periphery (skin and bone) showed higher infiltration of inflammatory cells. It contains fibroblasts, endothelial cells, and infiltrated inflammatory cells such as neutrophils, macrophages, and dendritic cells (Figure 1F,G).

### 3.2. SCC-PDSX Promotes the BMDCs Infiltration to the Tumor Invasion Front Line

We investigated the BMDCs infiltration by tracing the transplanted GFP-positive BM cells in the tumor. To examine the relationship of BMDCs and the invasiveness of different OSCC (VSCC and SCC), we quantified and compared the distribution of BMDCs in the invasion front line (peripheral area—bone and skin).

**Figure 2:**
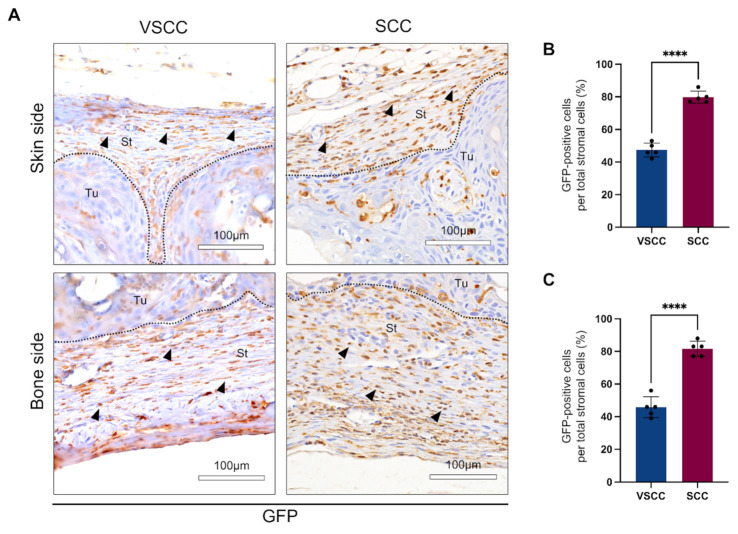
SCC-PDSX promotes GFP-positive BMDCs infiltration in the periphery of tumor: (**A**) Representative images of GFP IHC: upper panel: skin side; lower panel: bone side (left panel: VSCC-PDSX; right panel: SCC-PDSX); (**B**) The rate of infiltrating BMDCs into the skin side of tumors; (**C**) The rate of infiltrating BMDCs into the bone side of tumors. Dotted lines represent the boundary of the tumor (Tu) and the stroma (St). VSCC, verrucous oral squamous cells carcinoma; SCC, conventional oral squamous cell carcinoma, PDSX, patient-derived stromal cells xenograft. Statistical analyses were performed using Student’s *t*-test, 2-tailed; **** *p* < 0.0001. Dots in the plots represent the mean value of each mouse. Data are presented as the mean ± SD, *n* = 5.

In VSCC and SCC, GFP-positive cells were abundantly found in the periphery, and they were round, spindle, and dendritic in shape (Figure 2A). On the bone side, however, while most of the infiltrated GFP-positive cells in VSCC were spindle-shaped cells, most were round and dendritic in SCC. On quantification, infiltration of GFP-positive cells was significantly higher in SCC (approximately 80% of total stromal cells) than VSCC (approximately 45%) (*p* < 0.0001) (Figure 2B,C).

These data showed that the SCC-PDSX promoted the infiltration of bone marrow cells to the invasive front line, indicating the potential role of stromal cells in the relation of BMDCs to the invasiveness of OSCC.

### 3.3. MMP2-Positive BMDCs Infiltration Is Higher in the Invasive Front Line of SCC-PDX

Then, to identify the role of BMDCs in the invasion of OSCC, we examined the expression of MMP9 and MMP2 in the invasion front line of tumor specimens by performing IHC staining.

MMP9 staining showed the expression on the tumor cells of VSCC and SCC, especially in the periphery of the tumor nest. However, the stromal cells of both tumors did not express MMP9 (Figure S1).

**Figure 3:**
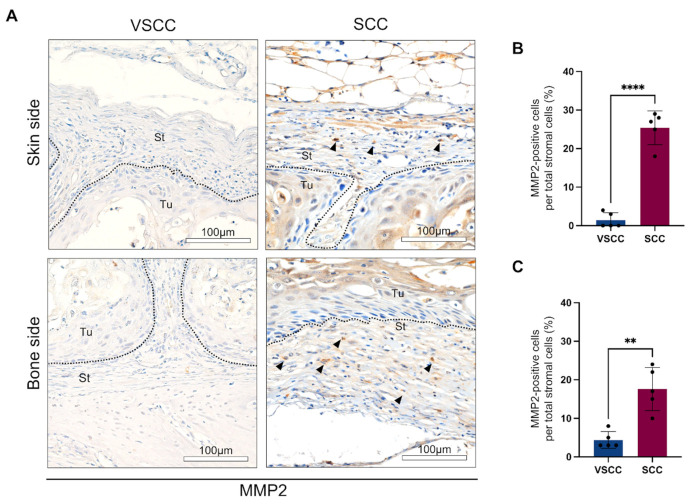
SCC-PDSX shows MMP2-positive cells’ infiltration in the periphery of tumor: (**A**) Representative images of MMP2 IHC: upper panel: skin side; lower panel: bone side (left panel: VSCC-PDSX; right panel: SCC-PDSX); (**B**) The rate of infiltrating MMP2-positive cells into the skin side of tumors; (**C**) The rate of infiltrating MMP2-positive cells into the bone side of tumors. Dotted lines represent the boundary of the tumor (Tu) and the stroma (St). VSCC, verrucous oral squamous cells carcinoma; SCC, conventional oral squamous cell carcinoma, PDSX, patient-derived stromal cells xenograft. Statistical analyses were performed using Student’s *t*-test, 2-tailed; ** *p* < 0.01, **** *p* < 0.0001. Dots in the plots represent the mean value of each mouse. Data are presented as the mean ± SD, *n* = 5.

Interestingly, MMP2 expression is different between VSCC and SCC. While VSCC showed the negative expression of MMP2 on tumor cells and a slight expression on stromal cells, SCC presented the high expression of MMP2 on both tumor and stroma areas (Figure 3A). MMP2 positive cells are spindle-shaped, rounded, and dendritic cells. They had a similar shape to GFP-positive cells. On quantification, we observed that MMP2 expression was significantly higher in the periphery of SCC than VSCC presenting 18–27% of stromal cells in SCC and less than 5% in VSCC (*p* < 0.0001 on skin side and *p* = 0.0012 on bone side) (Figure 3B,C).

**Figure 4:**
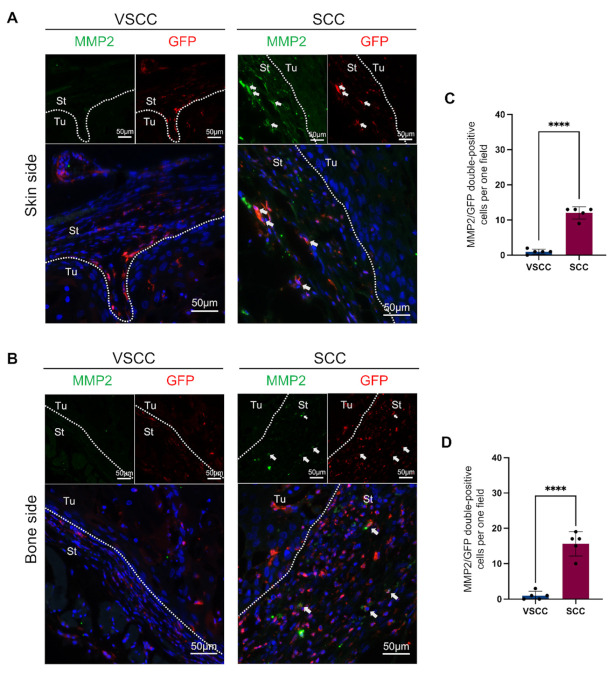
GFP/MMP2 double-positive BMDCs localized in the periphery of SCC-PDSX: (**A**) Representative images of GFP/MMP2 double-IHC staining on the skin side of tumors; (**B**) Representative images of GFP/MMP2 double-IHC staining at the skin side of tumors; (**C**) Comparison of GFP/MMP2 double-positive cells per one field (×400) in skin side of tumors; (**D**) Comparison of GFP/MMP2 double-positive cells per one field (×400) in bone side of tumors. Dotted lines represent the boundary of the tumor (Tu) and the stroma (St). VSCC, verrucous oral squamous cells carcinoma; SCC, conventional oral squamous cell carcinoma, PDSX, patient-derived stromal cells xenograft. Statistical analyses were performed using Student’s *t*-test, 2-tailed; **** *p* < 0.0001. Dots in the plots represent the mean value of each mouse. Data are presented as the mean ± SD, *n* = 5.

Furthermore, to confirm the MMP2 expression on BMDCs, we performed the double immunofluorescent staining on MMP2 and GFP. In the periphery, the co-expression of MMP2 and GFP is observed in the stromal area of SCC and rarely observed in VSCC (Figure 4A,B). MMP2/GFP double-positive cells are rounded or dendritic in shape. On quantification, MMP2-positive BMDCs are highly infiltrated in the peripheral area (both skin and bone side) in SCC than VSCC (Figure 4C,D). While 15–20 MMP2-positive cells per one magnified field (×400) were colocalized with GFP in SCC, VSCC showed 2–5 MMP2/GFP double-positive cells per one field (*p* < 0.0001). These findings indicated that BMDCs might participate in the invasion of OSCC by the expression of MMP2.

### 3.4. Stromal Factors IL6 and IL1B May Be Involved in the Relation of BMDCs and OSCC Invasion

**Figure 5:**
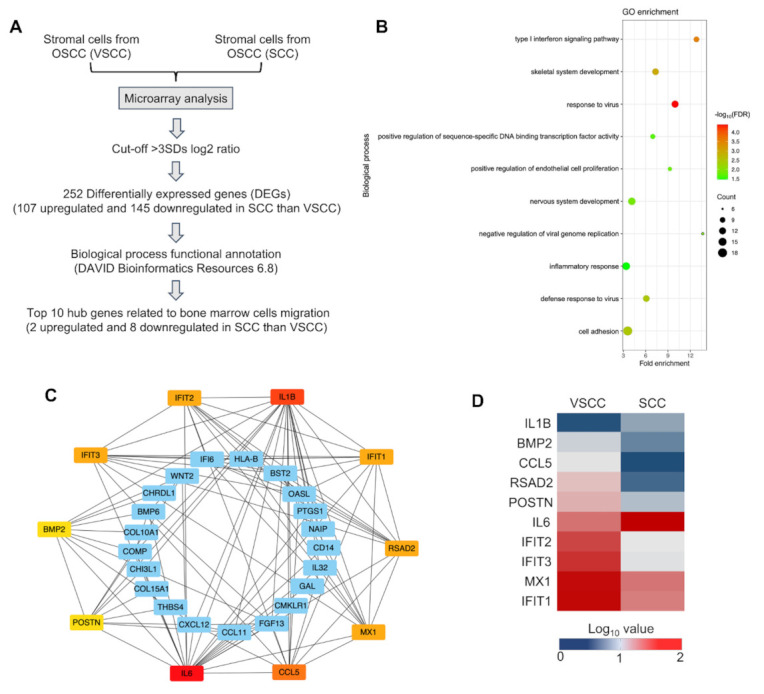
Identification of potential stromal factors involved in the relation of BMDCs and tumor invasion: (**A**) Flow chart of genes screening related to bone marrow cells migration; (**B**) The biological process enrichment analysis of common differentially expressed genes (DEGs) of VSCC stromal cells and SCC stromal cells; (**C**) Top 10 hub genes in bone marrow cells migration analyzed using protein-to-protein (PPI) network and Cytoscape software. (**D**) Heatmap presentation of the expression level of top 10 hub genes. VSCC, verrucous oral squamous cells carcinoma; SCC, conventional oral squamous cell carcinoma.

To elucidate the potential underlying mechanism of stromal factors in the involvement of BMDCs in OSCC invasion, DEGs in VSCC and SCC were sequentially analyzed and compared using a microarray (Figure 5A). First, we screened 252 DEGs which have more than 3SD in their expression level. We found that 107 genes were upregulated, and 145 genes were downregulated in SCC stromal cells than VSCC stromal cells (Tables S1 and S2). Furthermore, we identified the chemotaxis-related biological processes by using GO enrichment analysis (Figure 5B). Then, we performed the protein-to-protein interaction (PPI) network and selected the top 10 hub genes involved in the chemotaxis process as follows: interleukin 6 (*IL-6*), interleukin 1 beta (*IL1B*), bone morphogenetic protein 2 (*BMP2*), C–C motif chemokine ligand 5 (*CCL5*), radical S-adenosyl methionine domain containing 2 (*RSAD2*), periostin (*POSTN*), interferon-induced protein with tetratricopeptide repeats 1 (*IFIT1*), *IFIT2*, *IFIT3*, and MX dynamin-like GTPase 1 (*MX1*) (Figure 5C). Among these hub genes, *IL-6* mRNA and *IL1B* mRNA exhibited a higher expression in SCC stromal cells than VSCC stromal cells (Figure 5D). Therefore, stromal factors, *IL-6* and *IL1B* may underlie the different effects of SCC stromal cells and VSCC stromal cells in the relation of BMDCs and OSCC invasion.

## 4. Discussion

Tumor cells interplay with surrounding stroma and recruit BMDCs and create the favorable TME. There is accumulating evidence indicating that stroma-rich OSCC showed a poor prognosis [18,19] and the tumor stroma ratio is a promising prognostic tool for OSCC [20]. Tumor stroma components play an active role in tumor development and consist of heterogenous cells that can regulate tumor progression, invasion, and metastasis by producing growth factors and extracellular matrix [21,22,23]. Tumor and stroma cells recruit BMDCs into TME through the expression of chemokines such as CXC and CC and many other growth factors such as vascular endothelial growth factor (VEGF), transforming growth factor-β (TGF-β), and macrophage colony stimulating factor (M-CSF) [24]. BMDCs are capable of differentiating into different stromal cells such as CAFs, TECs, and TAMs and thus promote the tumorigenesis, tumor invasion, tumor angiogenesis, and immune suppression [11,13,25,26]. OSCC subtypes: VSCC and SCC show the difference in prognosis depending on the tumor invasiveness and metastasis. While SCC shows invasion, VSCC presents with less invasiveness and a better prognosis than SCC. Vast research has studied the differences such as angiogenesis, proliferative activity of SCC, and VSCC. [27]. However, the different contribution of stroma cells in OSCC subtypes has not been reported. In our study, we established the two different OSCC models which are GFP-positive bone marrow transplanted mice xenografted with different stroma cells isolated from VSCC (low invasion) and SCC (high invasion) and provided the potential role of stroma in the involvement of BMDCs in tumor invasion. Recently, the analysis of gene regulation in OSCC showed a promising target for diagnosis, treatment, and prognosis of OSCC [28]. However, the specific analysis of gene regulation in the stroma of OSCC subtypes is still unclear. Therefore, in this study, we analyzed the gene expression profile of stroma related to OSCC bone invasiveness.

Our study revealed that BMDCs accumulation is different according to the xenograft type. SCC-PDSX showed a higher accumulation of BMDCs in the periphery invasive area of the tumor than VSCC-PDSX. In cancer invasion, protease systems, including MMPs, are upregulated in both tumor and stromal cells at the primary tumor site. In oral cancer, as the tumor progresses, high MMP9 expression is observed in tumor epithelium and stroma and relates to poor prognosis [29]. However, our results showed that in VSCC- and SCC-PDSX, MMP9 expression was only observed in the tumor cells and was undetectable on BMDCs and stromal cells. In addition, MMP2 and MMP9 are also responsible for the recruitment of BMDCs, and recruited BMDCs amplify the BMDCs recruitment by the degradation of collagen IV via the expression of MMP2 [30]. Our data indicated that SCC-PDSX showed the expression of MMP2 on the infiltrated BMDCs, but VSCC-PDSX did not. These data suggested that MMP2-positive BMDCs might be the cause of the higher invasion of SCC.

Furthermore, we investigated the underlying reason for the difference in the recruitment of BMDCs between VSCC and SCC by microarray and bioinformatics analysis. The results indicated that *IL6* mRNA and *IL1B* mRNA were more upregulated in SCC than VSCC. It has been suggested that CAFs secrete IL6 promotes the metastasis and a key contributor to chemoresistance of gastric cancer cells [31,32]. IL6 also upregulates the expression of MMPs and promotes the migration and invasion of nasopharyngeal carcinoma cell lines [33]. Moreover, IL6 signaling promotes the invasion and metastasis of oral squamous cell carcinoma via the activation of integrin β1 [34]. However, the relation of IL6 expression by OSCC stroma and BMDCs infiltration to the invasive front line has not been extensively investigated. IL1B is expressed in a wide range of tissues and a variety of cells including macrophages, neutrophils, endothelial cells, and fibroblasts [35,36]. It has been reported that IL1B is highly expressed in solid cancers, including breast, colon, lung, head and neck cancers, and melanomas [37,38]. IL1B also plays a role in promoting the stemness and invasiveness of colon cancer cells [39]. However, to the best of our knowledge, no study to date has investigated the role of stroma secreted IL1B in the involvement of BMDCs in OSCC invasion. Future studies on the detailed investigation of the relation of stromal secreted IL-6 and IL1B in BMDCs involvement in tumor invasion will be of great interest for clinical application in the preoperative assessment of the cancer aggressiveness and anti-cancer therapies.

## 5. Conclusions

In conclusion, the present study demonstrated that the OSCC invasive subtype SCC stromal cell xenograft promoted the higher localization of BMDCs to the invasive front line of the tumor and the expression of MMP2 compared to the less-invasive subtype—the VSCC stromal cell xenograft. Finally, we observed that stromal factors such as IL-6 and IL1B may underlie the different effects of the involvement of MMP2-positive BMDCs in tumor invasion. These findings may provide potential insights into the three-factorial crosstalk of stromal factors, BMDCs recruitment, and OSCC invasion.

## Data Availability

The data are available in NCBI GEO (GSE164374) (https://www.ncbi.nlm.nih.gov/geo/query/acc.cgi?&acc=GSE164374, accessed on 10 November 2021). (The data is currently private and is scheduled to be released on 1 January 2024).

## Supplementary Materials

The following are available online at https://www.mdpi.com/article/10.3390/cancers14010137/s1, Figure S1: MMP9 expression in the stroma area of tumor periphery: upper panel: skin side; lower panel: Bone side (left panel: VSCC-PDSX; right panel: SCC-PDSX). Dotted lines represent the boundary of the tumor (Tu) and the stroma (St). VSCC, verrucous oral squamous cells carcinoma; SCC, conventional oral squamous cell carcinoma, PDSX, patient-derived stromal cells xenograft. Table S1: Upregulated genes with more than 3SD in SCC stromal cells than VSCC stromal cells. Table S2: Downregulated genes with more than 3SD in SCC stromal cells than VSCC stromal cells.
