# Supplementary material for: Cancer-Associated Stromal Cells Promote the Contribution of MMP2-Positive Bone Marrow-Derived Cells to Oral Squamous Cell Carcinoma Invasion
Source: Cancers (Basel). 2021 Dec 28;14(1):137. doi: 10.3390/cancers14010137 (PMC8750016; doi:10.3390/cancers14010137)
Supplement: Supplementary file 1 [file cancers-14-00137-s001.zip › cancers-1483923-Supplementary fixed.pdf]

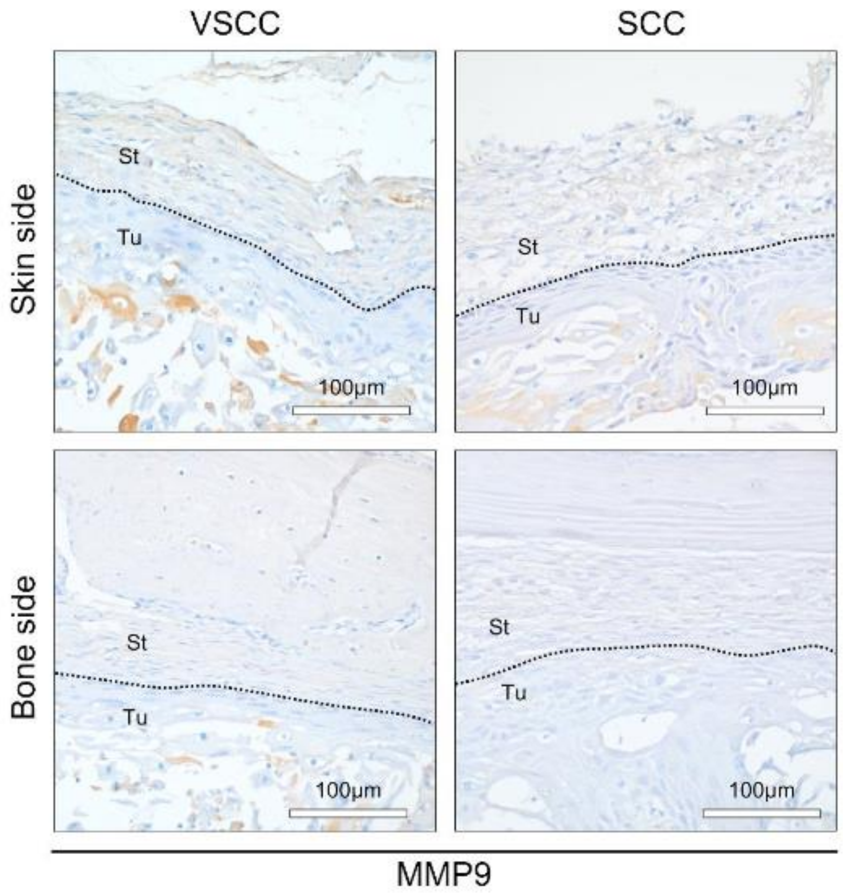

Figure S1. MMP9 expression in the stroma area of tumor periphery: Upper panel: skin side, Lower panel: Bone side (Left panel: VSCC-PDSX, Right panel: SCC-PDSX). Dotted lines represent the boundary of the tumor (Tu) and the stroma (St). VSCC, verrucous oral squamous cells carcinoma; SCC, conventional oral squamous cell carcinoma, PDSX, patient-derived stromal cells xenograft.

Upregulated genes in SCC stromal cells than VSCC stromal cells							
1	A_33_P3317460	31	ENST00000564390	61	lnc-ADCY9-1	91	SYNGR3
2	A_33_P3323914	32	ENST00000581935	62	lnc-BTBD10-3	92	SYTL2
3	A_33_P3422712	33	EPB41L3	63	lnc-SIK1-2	93	TES
4	ACP5	34	EPS8L1	64	LOC101927675	94	THNSL2
5	ACTG2	35	ESPNL	65	LOC101929494	95	TINAGL1
6	ADM2	36	FADS2	66	MMP1	96	TMEM130
7	ALDOC	37	FAIM2	67	MTHFD2	97	TMEM26-AS1
8	ARHGAP26	38	FAM49A	68	MTMR9LP	98	TNNT1
9	ASNS	39	FLT1	69	NAIP	99	TOX2
10	BCAT1	40	FNDC1	70	NR4A3	100	TRIB3
11	C17orf67	41	GAL	71	NR5A2	101	TRIM52-AS1
12	C19orf33	42	GFRA1	72	PAFAH1B3	102	TSPAN2
13	CBS	43	GPC3	73	PAX9	103	TUBB2B
14	CCDC8	44	GSTM5	74	PCDHB2	104	UCP2
15	CCL11	45	HCLS1	75	PCK2	105	WNT2
16	CEND1	46	HMHA1	76	PHGDH	106	XLOC_l2_014504
17	CHN1	47	IL1B	77	PITX1	107	XPNPEP3
18	CMKLR1	48	IL20RB	78	PRSS3		
19	COL15A1	49	IL32	79	PRSS3P2		
20	CXCL12	50	IL6	80	PSAT1		
21	DDIT4	51	KCNG1	81	RCOR2		
22	DOK7	52	KCNK12	82	S100A2		
23	DPYSL4	53	KRBOX1	83	SAA1		
24	ENPP1	54	LAMB3	84	SCD		
25	ENST00000438158	55	LAMC2	85	SFN		
26	ENST00000453852	56	LIMCH1	86	SHROOM3		
27	ENST00000511103	57	LINC00958	87	SLC6A9		
28	ENST00000518311	58	LINC01133	88	SMIM1		
29	ENST00000528497	59	LINC01444	89	STAT6		
30	ENST00000554254	60	LMNTD2	90	SUSD3		

Table S1. Upregulated genes with more than 3SD in SCC stromal cells than VSCC stromal cells.

Downregulated genes in SCC stromal cells than VSCC stromal cells							
1	ABCB4	38	ENST00000451118	75	lnc-C20orf96-4	112	PTGS1
2	ACKR3	39	ENST00000522356	76	lnc-CLVS1-1	113	QPCT
3	ADAMTS1	40	ETV1	77	lnc-DEC1-3	114	RGCC
4	ADAMTS19	41	EXTL1	78	lnc-FBXO25-3	115	RGS9
5	ADCY4	42	FAM19A5	79	lnc-MSRB3-2	116	RIMS1
6	ANGPTL4	43	FAM20A	80	lnc-RHPN1-2	117	RNF157
7	AOX1	44	FENDRR	81	lnc-SCRN3-2	118	RSAD2
8	ARL4C	45	FGF13	82	LOC100507002	119	SCIN
9	ATP8B4	46	FOLR3	83	LOC101926943	120	SEMA3A
10	BEX1	47	FRMPD4	84	LPPR4	121	SEMA5A
11	BIRC7	48	GABBR2	85	LRRN3	122	SHOX2
12	BMP2	49	GALNT15	86	MEDAG	123	SLC16A6
13	BMP6	50	GDF10	87	MGAT3	124	SLC24A3
14	BST2	51	GLDN	88	MMP12	125	SLC27A6
15	C15orf48	52	GRIA3	89	MOK	126	SNAP25
16	CASP10	53	GS1-259H13.2	90	MOXD1	127	SVEP1
17	CCDC85A	54	GSC	91	MX1	128	SVIL
18	CCL5	55	HERC5	92	NAP1L2	129	TCF21
19	CD14	56	HLA-B	93	NCKAP5	130	THBD
20	CFD	57	HLA-J	94	NEFL	131	THBS4
21	CHI3L1	58	IFI44L	95	NEFM	132	TM4SF1
22	CHRDL1	59	IFI6	96	NOVA1	133	TMEM158
23	CMPK2	60	IFIH1	97	NPR3	134	TMEM176A
24	COL10A1	61	IFIT1	98	NPTX1	135	TMEM176B
25	COMP	62	IFIT2	99	NSG1	136	TNXB
26	CRNDE	63	IFIT3	100	NXF3	137	TUBA4A
27	CYTL1	64	IGFBP2	101	OASL	138	VEPH1
28	DDX58	65	ITGB8	102	OLFM2	139	WBSCR27
29	DENND2A	66	KCND2	103	PAPPA	140	WNT16
30	DIO3	67	KRTAP1-5	104	PDE1C	141	WWC1
31	DIRC3	68	LCE2A	105	PDK4	142	ZDHHC11
32	DOK6	69	LCE2C	106	PLAU	143	ZFHX4
33	DPP9-AS1	70	LINC00842	107	PNMAL1	144	ZNF365
34	EDIL3	71	LINC01111	108	PNPLA3	145	ZNF385D
35	EDN1	72	LINC01291	109	PODXL		
36	ENST00000414768	73	lnc-ARSJ-1	110	POSTN		
37	ENST00000423433	74	lnc-BDKRB1-1	111	PTGDS		

Table S2. Downregulated genes with more than 3SD in SCC stromal cells than VSCC stromal cells.
